# The impact of peritoneal dialysis-related peritonitis on mortality in peritoneal dialysis patients

**DOI:** 10.1186/s12882-017-0588-4

**Published:** 2017-06-05

**Authors:** Hongjian Ye, Qian Zhou, Li Fan, Qunying Guo, Haiping Mao, Fengxian Huang, Xueqing Yu, Xiao Yang

**Affiliations:** 1 Department of Nephrology, The First Affiliated Hospital, Sun Yat-sen University, 58th, Zhongshan Road II, Guangzhou, 510080 China; 2 Key Laboratory of Nephrology, Ministry of Health, Guangzhou, 510080 China; 3 Guangdong Provincial Key Laboratory of Nephrology, Guangzhou, 510080 China; 4 Epidemiology Research Unit, The First Affiliated Hospital, Sun Yat-sen University, 58th, Zhongshan Road II, Guangzhou, 510080 China

**Keywords:** Peritoneal dialysis, Peritonitis, Mortality, Time-dependent variable

## Abstract

**Background:**

Results concerning the association between peritoneal dialysis-related peritonitis and mortality in peritoneal dialysis patients are inconclusive, with one potential reason being that the time-dependent effect of peritonitis has rarely been considered in previous studies. This study aimed to evaluate whether peritonitis has a negative impact on mortality in a large cohort of peritoneal dialysis patients. We also assessed the changing impact of peritonitis on patient mortality with respect to duration of follow-up.

**Methods:**

This retrospective cohort study included incident patients who started peritoneal dialysis from 1 January 2006 to 31 December 2011. Episodes of peritonitis were recorded at the time of onset, and peritonitis was parameterized as a time-dependent variable for analysis. We used the Cox regression model to assess whether peritonitis has a negative impact on mortality.

**Results:**

A total of 1321 patients were included. The mean age was 48.1 ± 15.3 years, 41.3% were female, and 23.5% with diabetes mellitus. The median (interquartile) follow-up time was 34 (21–48) months. After adjusting for confounders, peritonitis was independently associated with 95% increased risk of all-cause mortality (hazard ratio, 1.95; 95% confidence interval: 1.46–2.60), 90% increased risk of cardiovascular mortality (hazard ratio, 1.90; 95% confidence interval: 1.28–2.81) and near 4-fold increased risk of infection-related mortality (hazard ratio, 4.94; 95% confidence interval: 2.47–9.86). Further analyses showed that peritonitis was not significantly associated with mortality within 2 years of peritoneal dialysis initiation, but strongly influenced mortality in patients dialysed longer than 2 years.

**Conclusions:**

Peritonitis was independently associated with higher risk of all-cause, cardiovascular and infection-related mortality in peritoneal dialysis patients, and its impact on mortality was more significant in patients with longer peritoneal dialysis duration.

**Electronic supplementary material:**

The online version of this article (doi:10.1186/s12882-017-0588-4) contains supplementary material, which is available to authorized users.

## Background

The number of patients with end-stage renal disease (ESRD) who receive peritoneal dialysis (PD) therapy has been increasing worldwide [1] because of the improvement in PD techniques and concomitant patient survival. PD-related peritonitis remains the leading cause of technique failure in PD therapy [2, 3]. However, data regarding the impact of peritonitis on mortality in PD patients are controversial [4–9].

Some studies showed that peritonitis was associated with an increased risk of all-cause mortality in PD patients [4, 6, 7], although this was not confirmed by others [5, 8]. More recently, Hsieh et al. reported that patients with peritonitis had a lower risk of overall mortality in comparison with peritonitis-free patients [9]. The explanations for these confusing results may be related to the difficulty in evaluating statistically the relationship between peritonitis and mortality, especially with regard to the time-dependent effect of peritonitis; specifically, that patients who survive longer on PD carry a higher risk of experiencing a peritonitis event. The fact that peritonitis onset in patients with a longer duration of PD was related to poor outcomes [10, 11] also indicates that there is potentially time-dependent effect of peritonitis on mortality. To our knowledge, few studies have considered this time-dependent effect in their statistical methods when evaluating the influence of peritonitis on mortality in PD patients [4–6, 8, 9].

Therefore, in this study we evaluated whether peritonitis has a negative impact on mortality in a large cohort of PD patients, with peritonitis parameterized as a time-dependent variable. We also assessed the changing impact of peritonitis on patient mortality with respect to duration of follow-up.

## Methods

### Study population

This was a retrospective cohort study. Incident PD patients at the PD centre of The First Affiliated Hospital, Sun Yat-sen University, from 1 January 2006 to 31 December 2011 were included. Patients who were younger than 18 years, dropped out from PD within 90 days, or on long-term hemodialysis or had chronic renal transplant failure before initiating PD, were excluded. Tenckhoff catheters were placed using a sterile surgical technique and conventional PD solutions (Dianeal 1.5%, 2.5% or 4.25% dextrose; Baxter Healthcare, Guangzhou, China), and Y-sets and twin-bag systems were utilized in over 98% of the PD patients. Patients and their caregivers underwent a standard training program after catheterization. The study protocol was consistent with the ethical principles of the Helsinki Declaration and was approved by the Ethics Committee of The First Affiliated Hospital, Sun Yat-sen University. All participants were asked for permission to use their medical data for a non-commercial study, and written informed consent was obtained from them at the initiation of PD follow-up.

### Follow-up and outcomes

Demographic data, including age, sex, causes of ESRD, and comorbidity conditions, were collected at the time of PD initiation. A history of cardiovascular disease (CVD), which included cerebrovascular disease, ischemic heart disease, congestive heart failure, and peripheral vascular disease, was also reviewed. The baseline laboratory data were obtained during the first 1–3 months after initiating PD. All of the patients were followed up until death, or administrative censoring (including renal transplantation, switching to hemodialysis, transfer to another PD centre, loss to follow-up, and withdrawal of treatment), or the end of the study follow-up period (31 December 2013). Therefore, deaths while on PD treatment were considered as mortality in this study. The primary outcome was all-cause mortality. Secondary outcomes were infection-related mortality and cardiovascular (CV) mortality.

### Study definitions

PD-related peritonitis was diagnosed based on at least two of the following criteria [3]: (1) abdominal pain or cloudiness of PD effluent; (2) white blood cell count in PD effluent >100/μL with >50% polymorphonuclear leukocytes; and (3) a positive culture from PD effluent. CV death was defined as death due to ischemic heart disease, arrhythmias, sudden cardiac death, congestive heart failure, other heart disease, or cerebrovascular events [12, 13]. Death related to peritonitis was defined as death of a patient with active peritonitis, or admitted with peritonitis or within 2 weeks of a peritonitis episode [3]. When discussing the causes of deaths, the PD team, which consists of three nephrologists in our PD centre, reviewed the details of the individual medical records. Regarding inherent difficulties in assigning cause of death to infection or cardiovascular events, we tended to assign the death to the primary reason for hospitalization when the two coexisted.

#### Statistical analysis

Results were expressed as frequencies and percentages for categorical variables, mean ± standard deviation (SD) for continuous variables, and median and interquartile range (IQR) for skewed distributions. Probability of the cumulative rate of peritonitis episodes and mortality were evaluated by the Kaplan–Meier method. The proportional hazards assumption was tested using time-dependent covariates in the Cox regression model. When a peritonitis event was entered into the model as a conventional binary covariate, the proportional hazards assumption was violated (Additional file 1: Table S1), indicating peritonitis event as a conventional binary predictor was not appropriate for analyzing the effect of peritonitis event on survival. And if we considered only the first peritonitis in the Cox model for the study outcomes, the models were different (Additional file 1: Table S2). Thus, the onset time of all the episodes of peritonitis were recorded, and peritonitis was parameterized as a time-dependent variable (Additional file 1: Table S3), corresponding to the method suggested by published report [14]. For patients with peritonitis events, the time from the start of following-up to the time of peritonitis onset and the time from peritonitis onset to the death event were both calculated, respectively. The associations between peritonitis and all-cause, infection-related, and CV mortality were then assessed using Cox proportional hazards regression models. The potential confounders, including age, sex, diabetes mellitus (DM), history of CVD, 24-h urine output, hemoglobin, serum phosphorus, and serum albumin, some of which were identified as risk factors for mortality in our previous study [15], were also adjusted in the multivariable COX regression models.

Considering that the effects of peritonitis on mortality may be changing over the follow-up period, we analysed their associations progressively by year in the Cox regression models; meanwhile, a stratified analysis by follow-up time period was also performed to distinguish the differences. The statistical software SPSS (version 19.0; SPSS Inc. and IBM Inc.) was used for data analysis. All of the reported *p* values are two-tailed, and statistical significance was defined as *p* < 0.05.

## Results

### Study population

**Fig. 1:**
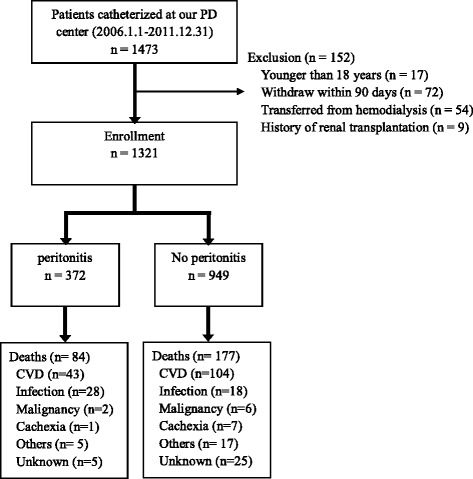
Flow chart for the study participants enrollment and outcomes. Abbreviations: CVD, cardiovascular disease; PD, peritoneal dialysis

A total of 1473 patients received PD therapy in our PD centre between 1 January 2006 and 31 December 2011. Patients who were younger than 18 years (*n* = 17), dropped out of PD within 90 days (*n* = 72), transferred from permanent hemodialysis (*n* = 54), and had a history of renal transplantation (*n* = 9) were excluded. A total of 1321 patients were ultimately included in the study (Fig. 1). The mean age of the entire cohort was 48.1 ± 15.3 years; 58.7% were male, 23.5% had DM, and 36.3% had a history of CVD. The demographic characteristics and clinical data of patients in the two cohorts are shown in Table 1.

**Table 1:** Demographic characteristics and clinical data

Variables	Patients (*n* = 1321)	Patients with peritonitis (*n* = 372)	Patients free of peritonitis (*n* = 949)	*P* value
Age (years)	48.1 ± 15.3	51.1 ± 14.7	46.9 ± 15.4	<0.001
Sex (Male, n, %)	776 (58.7%)	220 (59.1%)	556 (58.6%)	0.86
Primary renal disease (n, %)	-	-	-	0.006
Glomerulonephritis	776 (58.7%)	199 (53.5%)	577 (60.8%)	-
Diabetic nephropathy	298 (22.6%)	84 (22.6%)	214 (22.6%)	-
Hypertension	91 (6.9%)	38 (10.2%)	53 (5.6%)	-
Others	156 (11.8%)	51 (13.7%)	105 (11.1%)	-
Diabetes mellitus (n, %)	311 (23.5%)	87 (23.4%)	224 (23.6%)	0.93
History of CVD (n, %)	479 (36.3%)	139 (37.4%)	340 (35.8%)	0.62
Duration of PD (months)	34 (21–48)	40 (26–56)	32 (18–46)	<0.001
24-h urine output (mL)	900 (500–1300)	875 (500–1300)	900 (500–1300)	0.53
Hemoglobin (g/dL)	9.4 ± 2.3	9.2 ± 2.3	9.4 ± 2.3	0.133
Serum phosphorus (mg/dL)	5.2 ± 1.7	5.1 ± 1.7	5.2 ± 1.8	0.124
Serum albumin (g/dL)	3.6 ± 0.5	3.6 ± 0.5	3.7 ± 0.5	0.080

NOTE. Values expressed as mean ± SD, median (interquartile range), or number (percent); *CVD* cardiovascular disease; *PD* peritoneal dialysis

### Episodes of peritonitis

**Fig. 2:**
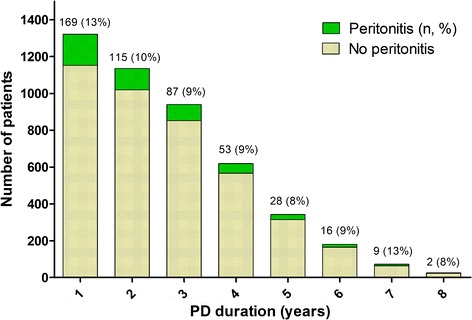
The proportion of patients experienced peritonitis events in different duration of peritoneal dialysis

**Fig. 3:**
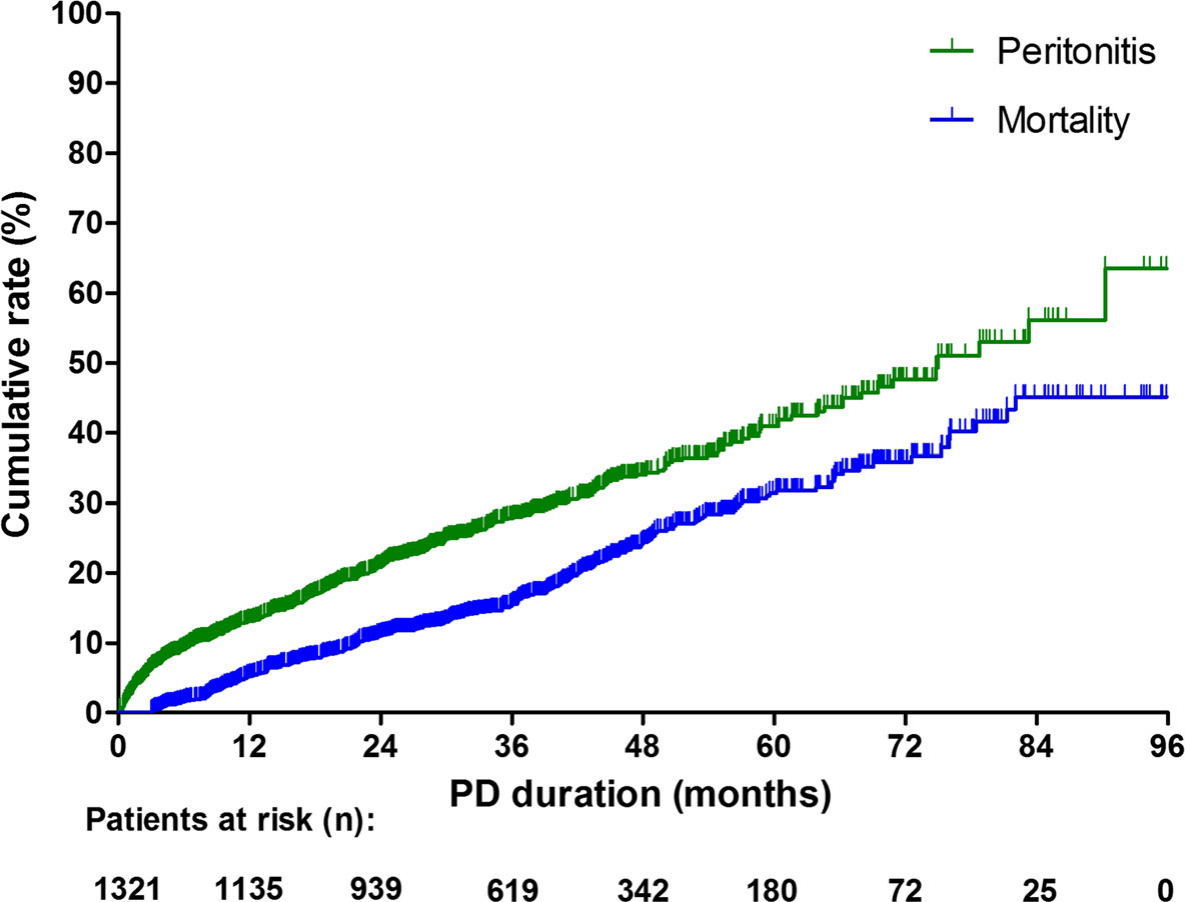
Cumulative risk of experiencing peritonitis events and all-cause mortality in all patients, estimated by Kaplan-meier survival analysis

During the median of 34 (IQR: 21–48) months of follow-up, 372 (28.2%) patients experienced episodes of peritonitis. Among them, 234 (62.9%) had one episode of peritonitis, 72 (19.4%) had two episodes, and 66 (5.5%) had three episodes or more (Table 2). The peritonitis rate was 0.16 per patient-year (95% confidence interval [CI] 0.14–0.18). Figure 2 shows the distribution of patients who experienced peritonitis by dialysis duration. In the first year of PD initiation, 169 (13%) patients had experienced episodes of peritonitis, and the proportion of patients with peritonitis fluctuated from 8% to 13% in the subsequent years. As estimated by Kaplan–Meier survival analysis, the cumulative risk of experiencing peritonitis events and all-cause mortality increased in concert with the extension of PD duration (Fig. 3).

**Table 2:** Peritonitis events and clinical outcomes at the end of the follow-up period for the entire cohort

Outcomes	Patients (*n* = 1321)	Follow-up time (months)
Peritonitis events (n, %)		
0 time	949 (71.8%)	32 (18–46)
1 time	234 (17.7%)	38 (25–55)
2 times	72 (5.5%)	45 (29–59)
≥3 times	66 (5.0%)	41 (26–58)
Clinical outcomes (n, %)		
Deaths	261 (19.8%)	24 (12–42)
Transferred to hemodialysis	111 (8.4%)	26 (15–44)
Transferred to renal transplantation	218 (16.5%)	17 (15–44)
Transferred to other PD centres	63 (4.8%)	26 (16–36)
Loss to follow up	42 (3.2%)	29 (18–39)
Withdrew treatment	14 (1.4%)	10 (8–16)
Renal function recovery	1 (0.1%)	5
Stay on PD	611 (46.3%)	45 (34–59)

NOTE. Values expressed as median (interquartile range), or number (percent)

*PD* peritoneal dialysis

### Associations between peritonitis and mortality

By the end of the follow-up period, 261 (19.8%) of the 1321 patients had died, 111 (8.4%) were transferred to hemodialysis, 218 (16.5%) received renal transplantation, and 611 (46.3%) remained on PD (Table 2). The mortality rate of the patients was 0.07 per patient-year (95% CI: 0.06–0.08). A total of 147 (56.3%) patients died due to cardiovascular disease while 46 (17.6%) died of infectious disease, of which 19 (41.3%) were peritonitis-related. The etiological details of all-cause deaths and infection-related deaths in the cohort are shown in Fig. 1 and Table 3, respectively. Similarly, the risks for peritonitis and death both increased as the PD duration extended (Fig. 2).

**Table 3:** Etiology of infection-related deaths

Causes	N (%)
Peritonitis	19 (41.3%)
Pneumonia	16 (34.8%)
Gastrointestinal infections	5 (10.9%)
Diabetic foot and subsequent sepsis	2 (4.3%)
Acute endocarditis	1 (2.2%)
Acute gallstone pancreatitis	1 (2.2%)
Tuberculosis	1 (2.2%)
Sepsis with pathogen from unclear foci	1 (2.2%)
Total	46 (100%)

As shown in Table 4, peritonitis was associated with an increased risk of all-cause mortality, infection-related mortality, and CV mortality in the study population. After adjustment for age, sex, DM, history of CVD, 24-h urine output, hemoglobin, serum phosphorus, and serum albumin, peritonitis was independently associated with a higher risk of all-cause mortality (hazard ratio [HR] = 1.95, 95% CI: 1.46–2.60, *p* < 0.001), infection-related mortality (HR = 4.94, 95% CI: 2.47–9.86, *p* < 0.001), and CV mortality (HR = 1.90, 95% CI: 1.28–2.81, *p* < 0.001).

**Table 4:** Associations between peritonitis and mortality using the COX proportional hazards regression models

	Univariate model		Multivariate model	
	HR (95% CI)	*P* value	HR (95% CI)	*P* value
All-cause mortality				
Peritonitis^a^	2.19 (1.68–2.85)	<0.001	1.95 (1.46–2.60)	<0.001
Age (per year increase)	1.07 (1.06–1.08)	<0.001	1.04 (1.03–1.05)	<0.001
Male gender	0.96 (0.75–1.23)	0.756	1.05 (0.80–1.37)	0.750
Diabetes mellitus	3.76 (2.95–4.80)	<0.001	1.97 (1.48–2.63)	<0.001
History of CVD	4.17 (3.23–5.39)	<0.001	2.01 (1.48–2.74)	<0.001
24-h urine output (per 100 ml increase)	0.93 (0.90–0.95)	<0.001	0.97 (0.94–0.99)	0.023
Hemoglobin (g/dL)	0.78 (0.73–0.82)	<0.001	0.83 (0.77–0.88)	<0.001
Serum phosphorus (mg/dL)	1.20 (1.13–1.28)	<0.001	1.16 (1.08–1.25)	<0.001
Serum albumin (g/dL)	0.39 (0.31–0.49)	<0.001	0.83 (0.64–1.07)	0.155
Infection-related mortality				
Peritonitis^a^	6.00 (3.26–11.03)	<0.001	4.94 (2.47–9.86)	<0.001
CV mortality				
Peritonitis^a^	2.05 (1.43–2.93)	<0.001	1.90 (1.28–2.81)	<0.001

^a^Peritonitis was parameterized as a time-dependent covariate. Multivariable models for peritonitis were adjusted for age, sex, diabetes, history of CVD, 24-h urine output, hemoglobin, serum phosphorus, and serum albumin.

*CVD* cardiovascular disease, *PD* peritoneal dialysis

For age was also independently associated with all-cause, infection-related, and CV mortality, we further examined the interaction effects between age and peritonitis on mortality. However, no statistically significant interaction effects were apparent (Additional file 1: Table S4).

### Changing effects of peritonitis on mortality throughout the follow-up period

**Fig. 4:**
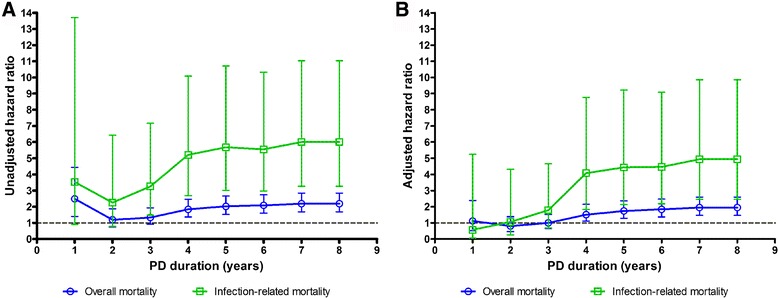
The crude hazard ratios (**a**) and adjusted hazard ratios (**b**) of peritonitis for all-cause and infection-related mortality over the follow-up times. Note: Adjusted hazard ratio for peritonitis event was adjusted for age, sex, diabetes, history of CVD, 24-h urine output, hemoglobin, serum phosphorus, and serum albumin in the multivariable COX regression models

The negative impacts of peritonitis on mortality changed by the follow-up period (Fig. 4). The adjusted HR of peritonitis for all-cause and infection-related mortality was 0.80 (95% CI: 0.46-1.38) and 1.06 (95% CI: 0.26-4.32) for patients within 2 years of PD initiation, respectively, gradually increasing to 1.74 (95% CI: 1.28-2.36) and 4.44 (95% CI: 2.14-9.22) after 5 years of PD treatment, the HRs sustained thereafter (for detailed data see Additional file 1: Table S5).

Based on the results shown in Fig. 4, we further conducted a stratified analysis by the follow-up time period in the Cox regression model. For patients within 2 years of PD, the adjusted HR of peritonitis for all-cause and infection-related mortality was 0.80 (95% CI: 0.46–1.38) and 1.06 (95% CI: 0.26–4.32), respectively, while it was much higher in patients on dialysis longer than 2 years, with an adjusted HR of 3.98 (95% CI: 2.70–5.86) and 9.33 (95% CI: 3.56–24.47), respectively. The interaction test also demonstrated that the relationship between peritonitis and follow-up period for all-cause and infection-related mortality was statistically significant (Table 5).

**Table 5:** Stratified analysis by follow-up time period for mortality for patients who experienced peritonitis in the COX proportional hazards regression models

Time period (years)	No. of patients	No. of deaths	Univariate model		Multivariate model^#^	
			HR (95% CI)	*P* value	HR (95% CI)	*P* value
All-cause mortality^a^						
Overall	1321	261	2.19 (1.68–2.85)	<0.001	1.95 (1.46–2.60)	<0.001
≤2 years	1321	131	1.19 (0.75–1.88)	0.465	0.80 (0.46–1.38)	0.421
>2 years	939	130	4.65 (3.26–6.63)	<0.001	3.98 (2.70–5.86)	<0.001
Infection-related mortality^b^						
Overall	1321	46	6.00 (3.26–11.03)	<0.001	4.94 (2.47–9.86)	<0.001
≤2 years	1321	16	2.25 (0.79–6.43)	0.129	1.06 (0.26–4.32)	0.932
>2 years	939	30	12.47 (5.18–30.01)	<0.001	9.33 (3.56–24.47)	<0.001
CV mortality^c^						
Overall	1321	147	2.05 (1.43–2.93)	<0.001	1.90 (1.28–2.81)	0.001
≤2 years	1321	75	1.05 (0.55–1.99)	0.882	0.69 (0.32–1.48)	0.339
>2 years	939	72	4.51 (2.80–7.27)	<0.001	4.13 (2.46–6.94)	<0.001

Note: Peritonitis was parameterized as a time-dependent covariate

^#^Multivariable models were adjusted for age, sex, diabetes, history of CVD, 24-h urine output, hemoglobin, serum phosphorus, and serum albumin.

^a^Overall test for interaction of peritonitis and time period, Wald ^2^ = 8.23, *P* = 0.004; all-cause mortality.

^b^Overall test for interaction of peritonitis and time period, Wald^2^ = 6.02, *P* = 0.014; infection-related mortality.

^c^Overall test for interaction of peritonitis and time period, Wald^2^ = 2.32, *P* = 0.128; CV mortality

### Discussion

In this cohort study covering 1321 incident PD patients, we demonstrated that peritonitis was independently associated with higher risk of all-cause mortality, infection-related mortality, and CV mortality. Further analysis showed that the impacts of peritonitis on mortality were more significant in patients with longer PD duration.

Controversial conclusions were made in previous studies regarding the impact of peritonitis on mortality in PD patients. Fried et al. found that an increased peritonitis rate was an independent risk factor for overall mortality in 516 adult PD patients in a single centre in Pittsburgh [4]. Boudville et al., using a case-crossover design, demonstrated that there was an approximately 6-fold increase in the odds of peritonitis in the 30 days before death compared with the 30-day window 6 months before death [7]. However, in a retrospective study of 565 PD patients in Spain, the peritonitis rate was not significantly associated with all-cause mortality after adjustment for potential confounders [5]. Moreover, Hsieh et al. reported recently that patients with a history of peritonitis episodes had a lower risk of all-cause mortality in a Kaplan–Meier analysis and multivariate Cox regression in a Taiwan population with a peritonitis rate of 0.196 episodes per patient-year [9]. These controversial conclusions may be attributed to the difficulty in evaluating the impact of peritonitis on mortality. First, at present there is no standard definition of peritonitis-related mortality. Second, the indirect long-term effects of peritonitis on mortality, which would be mediated by the inflammation state [16–18], poor nutritional status [17], and/or peritoneal membrane change after peritonitis events [19], may be obscure and difficult to define. Moreover, our preliminary exploration found that when a peritonitis event as a conventional binary covariate was entered into the time-dependent Cox regression model, the proportional hazards assumption was violated (Additional file 1: Table S1), suggesting that peritonitis event was a time-dependent covariate for mortality, which has rarely been considered in the statistical methods of most of the previous studies.

In observational studies, treatment is often time dependent. The time from the beginning of follow-up to treatment initiation, with no study event occurs, is known as immortal time in epidemiological studies, and mishandling immortal time can lead to an overestimated treatment effect, sometimes even draw an inverse conclusion [14]. Similar to the treatment, peritonitis is also time dependent. The time from the beginning of follow-up to the time of peritonitis onset should be treated as another kind of immortal time, which being properly handled will be extremely important when assessing the impacts of peritonitis on outcomes. Referring to the suggestions by the authors of the published article above [14], considering the treatment as time-varying variable in the COX regression model would be a prior choice for controlling the immortal time bias. Therefore, with peritonitis parameterizing as a time-dependent variable, we used the Cox regression models to assess the effects of peritonitis on mortality, and demonstrated that peritonitis was independently associated with a higher risk of mortality in our cohort of PD patients.

It is not difficult to understand the negative impact of peritonitis on mortality in PD patients. First, severe and/or persistent peritonitis may lead to serious complications such as intestinal obstruction, intestinal perforation and sepsis, which can directly cause death. Therefore, such severe and/or persistent peritonitis may be readily related to the cause of a PD patient’s mortality. Second, the indirect long-term effects of peritonitis on mortality, as already mentioned [16–19], have been emphasized in recent years. Moreover, patients who are prone to experience an episode of peritonitis may also be likely to experience other infections, as they may share similar risk factors. For example, lower serum albumin, which was verified as a remarkable risk factor for peritonitis episodes in PD patients [20–22], was also demonstrated to be an independent risk factor for pneumonia in our centre’s previous work [23]. As also shown in this study, patients with peritonitis episodes had a significantly higher risk for infection-related mortality, although peritonitis events only accounted for 41.3% of the direct causes of infection-related deaths.

We also found that the negative effects of peritonitis on mortality increased among patients with longer PD follow-up times. Previous studies have reported that the first episodes of peritonitis had better outcomes than subsequent ones, and that a longer duration of PD was associated with worse clinical outcomes of peritonitis [5, 10, 11, 24], which may partially support our findings. In the study conducted by Xu et al., among patients with subsequent peritonitis episodes, those with longer PD duration had a significantly higher drop-out (defined as death or transferring to hemodialysis) rate than those with shorter duration (30.6% vs. 9.7%) [11]. In addition, Krishnan et al. have reported a higher non-resolution rate (24.4% vs. 16.5%) of peritonitis in patients on PD for more than 2.4 years than those on PD for less than 2.4 years [25]. In comparison with these studies, our findings did not directly demonstrate that PD duration affected the impact of peritonitis on mortality, but did indicate that the risk for mortality increased in patients with longer PD duration who experienced a peritonitis event. The mechanisms of this phenomenon remain unclear. On the one hand, the impaired host defence due to the long-term exposure of the conventional glucose dialysate and malnutrition [26–29] may be the main contributor to the poor outcomes in long-term PD patients when exposed to an episode of peritonitis, although this notion needs to be clarified. On the other hand, a longer duration of PD may also be associated with chronic inflammation [30–32] and cardiovascular calcification [33, 34], which are risk factors for overall and CV mortality in PD patients. In addition, ESRD patients on dialysis for a number of years were associated with higher levels of alkaline phosphatase [35, 36], which is traditionally a marker of high-turnover bone disease in ESRD patients but was recently found to be an independent predictor of adverse outcomes of peritonitis in PD patients [35].

## Conclusions

The strengths of this study include the large sample size, and the complete records of peritonitis and death events in the cohort. In addition, the methodology we used had adequately dealt with the time-dependent feature of peritonitis. However, there are some limitations. First, as all patients included were from a single PD centre, selection bias cannot be excluded, although the patients were from diverse districts of southern China. Second, as this was a retrospective study, some potential confounding effects could not be completely discounted. The third, this study could not answer the question that how long the negative effect of peritonitis would last on mortality, which probably varies in each patient. Finally, the number of patients with unknown causes of death in the group free of peritonitis was much higher than that in the peritonitis group, and such an unequal distribution may interfere with the balance of observations.

In conclusion, peritonitis was independently associated with a higher risk of all-cause, infection-related and cardiovascular mortality, in those patients on peritoneal dialysis longer than 2 years who experienced a peritonitis. Given the poor outcome of peritonitis in the long-term PD patients, reducing the incidence rate of peritonitis remains the most important challenge in the management of this population of patients.

## Additional files

Additional file 1: Table S1. The PH assumption for the assessment of the relationship between peritonitis and all cause mortality in peritoneal dialysis patients. **Table S2.** The different models if considering only the first peritonitis in the Cox model for the study outcomes. **Table S3.** Illustration for the follow-up time and how to parameterize peritonitis as a time-dependent for analysis in the COX regression model. **Table S4.** The interactions between age and peritonitis for the assessment of the risk for mortality. **Table S5.** The HRs of peritonitis for all-cause and infection-related mortality progressively by year in the COX proportional hazards models (DOC 97 kb)

## Abbreviations

CI: Confidence interval
CV: Cardiovascular
CVD: Cardiovascular disease
DM: Diabetes mellitus
ESRD: End-stage renal disease
HR: Hazard ratio
IQR: Interquartile range
PD: Peritoneal dialysis;
SD: Standard deviation

## References

[1] Liu FX, Gao X, Inglese G, Chuengsaman P, Pecoits-Filho R, Yu A (2014). A global overview of the impact of peritoneal dialysis first or favored policies: an opinion. Perit Dial Int.

[2] Troidle L, Gorban-Brennan N, Kliger A, Finkelstein FO (2003). Continuous peritoneal dialysis-associated peritonitis: a review and current concepts. Semin Dial.

[3] Li PK, Szeto CC, Piraino B, Bernardini J, Figueiredo AE, Gupta A (2010). Peritoneal dialysis-related infections recommendations: 2010 update. Perit Dial Int.

[4] Fried LF, Bernardini J, Johnston JR, Piraino B (1996). Peritonitis influences mortality in peritoneal dialysis patients. J Am Soc Nephrol.

[5] Perez Fontan M, Rodriguez-Carmona A, Garcia-Naveiro R, Rosales M, Villaverde P, Valdes F (2005). Peritonitis-related mortality in patients undergoing chronic peritoneal dialysis. Perit Dial Int.

[6] Sipahioglu MH, Aybal A, Unal A, Tokgoz B, Oymak O, Utas C (2008). Patient and technique survival and factors affecting mortality on peritoneal dialysis in Turkey: 12 years' experience in a single center. Perit Dial Int.

[7] Boudville N, Kemp A, Clayton P, Lim W, Badve SV, Hawley CM (2012). Recent peritonitis associates with mortality among patients treated with peritoneal dialysis. J Am Soc Nephrol.

[8] Klaric D, Knotek M (2013). Long-term effects of peritonitis on peritoneal dialysis outcomes. Int Urol Nephrol.

[9] Hsieh YP, Chang CC, Wen YK, Chiu PF, Yang Y (2014). Predictors of peritonitis and the impact of peritonitis on clinical outcomes of continuous ambulatory peritoneal dialysis patients in Taiwan--10 years' experience in a single center. Perit Dial Int.

[10] Yi C, Yang X, Guo Q, Jiang Z, Lin J, Cai J (2011). The clinical features of first episode of peritonitis in patients with long-term peritoneal dialysis. Chin J Nephrol.

[11] Xu R, Chen Y, Luo S, Li Y, Dong J (2012). The influence of duration of peritoneal dialysis therapy on the outcomes of initial and subsequent peritonitis is different. Perit Dial Int.

[12] Cheung AK, Sarnak MJ, Yan G, Berkoben M, Heyka R, Kaufman A (2004). Cardiac diseases in maintenance hemodialysis patients: results of the HEMO study. Kidney Int.

[13] Delmez JA, Yan G, Bailey J, Beck GJ, Beddhu S, Cheung AK (2006). Cerebrovascular disease in maintenance hemodialysis patients: results of the HEMO study. Am J Kidney Dis.

[14] Liu J, Weinhandl ED, Gilbertson DT, Collins AJ, St Peter WL (2012). Issues regarding 'immortal time' in the analysis of the treatment effects in observational studies. Kidney Int.

[15] Liu X, Huang R, Wu H, Wu J, Wang J, Yu X (2016). Patient characteristics and risk factors of early and late death in incident peritoneal dialysis patients. Sci Rep.

[16] Lai KN, Lai KB, Lam CW, Chan TM, Li FK, Leung JC (2000). Changes of cytokine profiles during peritonitis in patients on continuous ambulatory peritoneal dialysis. Am J Kidney Dis.

[17] Lam MF, Leung JC, Lo WK, Tam S, Chong MC, Lui SL (2007). Hyperleptinaemia and chronic inflammation after peritonitis predicts poor nutritional status and mortality in patients on peritoneal dialysis. Nephrol Dial Transplant.

[18] Zalunardo NY, Rose CL, Ma IW, Altmann P (2007). Higher serum C-reactive protein predicts short and long-term outcomes in peritoneal dialysis-associated peritonitis. Kidney Int.

[19] van Diepen AT, van Esch S, Struijk DG, Krediet RT (2014). The first peritonitis episode alters the natural course of peritoneal membrane characteristics in peritoneal dialysis patients. Perit Dial Int.

[20] Fan X, Huang R, Wang J, Ye H, Guo Q, Yi C (2014). Risk factors for the first episode of peritonitis in southern Chinese continuous ambulatory peritoneal dialysis patients. PLoS One.

[21] Wang Q, Bernardini J, Piraino B, Fried L (2003). Albumin at the start of peritoneal dialysis predicts the development of peritonitis. Am J Kidney Dis.

[22] Chow KM, Szeto CC, Leung CB, Kwan BC, Law MC, Li PK (2005). A risk analysis of continuous ambulatory peritoneal dialysis-related peritonitis. Perit Dial Int.

[23] He F, Wu X, Xia X, Peng F, Huang F, Yu X (2013). Pneumonia and mortality risk in continuous ambulatory peritoneal dialysis patients with diabetic nephropathy. PLoS One.

[24] Szeto CC, Kwan BC, Chow KM, Law MC, Pang WF, Chung KY (2009). Recurrent and relapsing peritonitis: causative organisms and response to treatment. Am J Kidney Dis.

[25] Krishnan M, Thodis E, Ikonomopoulos D, Vidgen E, Chu M, Bargman JM (2002). Predictors of outcome following bacterial peritonitis in peritoneal dialysis. Perit Dial Int.

[26] Hekking LH, Zareie M, Driesprong BA, Faict D, Welten AG, de Greeuw I (2001). Better preservation of peritoneal morphologic features and defense in rats after long-term exposure to a bicarbonate/lactate-buffered solution. J Am Soc Nephrol.

[27] Wu J, Yang X, Zhang YF, Wang YN, Liu M, Dong XQ (2010). Glucose-based peritoneal dialysis fluids downregulate toll-like receptors and trigger hyporesponsiveness to pathogen-associated molecular patterns in human peritoneal mesothelial cells. Clin Vaccine Immunol.

[28] Jones S, Holmes CJ, Mackenzie RK, Stead R, Coles GA, Williams JD (2002). Continuous dialysis with bicarbonate/lactate-buffered peritoneal dialysis fluids results in a long-term improvement in ex vivo peritoneal macrophage function. J Am Soc Nephrol.

[29] Pajek J, Gucek A, Kveder R, Bucar-Pajek M, Kaplan-Pavlovcic S, Bren AF (2008). Impact of dialysis duration and glucose absorption on nutritional indices in stable continuous ambulatory peritoneal dialysis patients. J Ren Nutr.

[30] Ates K, Ates A, Ekmekci Y, Nergizoglu G (2005). The time course of serum C-reactive protein is more predictive of mortality than its baseline level in peritoneal dialysis patients. Perit Dial Int.

[31] Stenvinkel P, Lindholm B, Lonnqvist F, Katzarski K, Heimburger O (2000). Increases in serum leptin levels during peritoneal dialysis are associated with inflammation and a decrease in lean body mass. J Am Soc Nephrol.

[32] Pecoits-Filho R, Stenvinkel P, Wang AY, Heimburger O, Lindholm B (2004). Chronic inflammation in peritoneal dialysis: the search for the holy grail?. Perit Dial Int.

[33] Shantouf R, Kovesdy CP, Kim Y, Ahmadi N, Luna A, Luna C (2009). Association of serum alkaline phosphatase with coronary artery calcification in maintenance hemodialysis patients. Clin J Am Soc Nephrol.

[34] Stompor TP, Pasowicz M, Sulowicz W, Dembinska-Kiec A, Janda K, Wojcik K (2004). Trends and dynamics of changes in calcification score over the 1-year observation period in patients on peritoneal dialysis. Am J Kidney Dis.

[35] Ye H, Lin X, Qiu Y, Guo Q, Huang F, Yu X (2015). Higher alkaline phosphatase was associated with the short-term adverse outcomes of peritoneal dialysis-related peritonitis. Clin Chem Lab Med.

[36] Beddhu S, Baird B, Ma X, Cheung AK, Greene T (2010). Serum alkaline phosphatase and mortality in hemodialysis patients. Clin Nephrol.

