# Supplementary material for: The impact of peritoneal dialysis-related peritonitis on mortality in peritoneal dialysis patients
Source: BMC Nephrol. 2017 Jun 5;18:186. doi: 10.1186/s12882-017-0588-4 (PMC5460447; doi:10.1186/s12882-017-0588-4)
Supplement: Supplementary file 1 — The PH assumption for the assessment of the relationship between peritonitis and all cause mortality in peritoneal dialysis patients. Table S2. The different models if considering only the first peritonitis in the Cox model for the study outcomes. Table S3. Illustration for the follow-up time and how to parameterize peritonitis as a time-dependent for analysis in the COX regression model. Table S4. The interactions between age and peritonitis for the assessment of the risk for mortality. Table S5. The HRs of peritonitis for all-cause and infection-related mortality progressively by year in the COX proportional hazards models (DOC 97 kb) [file 12882_2017_588_MOESM1_ESM.doc]

**The impact of peritoneal dialysis-related peritonitis on mortality in peritoneal dialysis patients**

Hongjian Ye^1-3*^ MD, Qian Zhou^1-4*^ MD, Li Fan^1-3^ MD & PhD, Qunying Guo^1-3^ MD & PhD, Haiping Mao^1-3^ MD & PhD, Fengxian Huang^1-3^ MD & PhD, Xueqing Yu^1-3^ MD & PhD, Xiao Yang^1-3#^ MD & PhD

^1^ Department of Nephrology, The First Affiliated Hospital, Sun Yat-sen University, 58th, Zhongshan Road II, Guangzhou, 510080, China

^2^ Key Laboratory of Nephrology, Ministry of Health, Guangzhou, 510080, China

^3^ Guangdong Provincial Key Laboratory of Nephrology, Guangzhou, 510080, China

^4^ Epidemiology Research Unit, The First Affiliated Hospital, Sun Yat-sen University, 58th, Zhongshan Road II, Guangzhou, 510080, China

* These authors contribute equally to this work.

^#^ Correspondent author: Xiao Yang, MD, PhD.

Department of Nephrology, The First Affiliated Hospital, Sun Yat-sen University, Guangzhou 510080, China.

Phone: 0086-20-87755766-8843. Fax: 0086-20-87769673.

E-mail address: yangxsysu@126.com

**Supplementary information**

**Supplementary Table S1. The PH assumption for the assessment of the relationship between peritonitis and overall mortality in peritoneal dialysis patients**

|  | **Hazard ratio** | **95% confident interval** | **P value** |
| --- | --- | --- | --- |
| T_cov_^#^ | 1.81 | 1.23-2.65 | 0.002 |
| Peritonitis* | 0.14 | 0.04-0.50 | 0.003 |

^#^T_cov_ was expressed as: LN (T_) * peritonitis;

*peritonitis was analyzed as a conventional binary covariate grouped by whether the patient experienced peritonitis event during the follow-up period.

**Supplementary Table S2. The different models if considering only the first peritonitis in the Cox model for the study outcomes**

|  | **Univariate model** | |
| --- | --- | --- |
|  | HR (95% CI) | P value |
| **All-cause mortality** |  |  |
| Peritonitis* | 0.92 (0.71-1.19) | 0.526 |
| **Infection-related mortality** |  |  |
| Peritonitis* | 2.91 (1.60-5.29) | <0.001 |
| **CV mortality** |  |  |
| Peritonitis* | 0.80 (0.56-1.14) | 0.216 |

Note: Peritonitis* was considered only the first peritonitis in the Cox regression model.

**Supplementary Table S3. Illustration for the follow-up time and how to parameterize** peritonitis as a time-dependent for analysis in the COX regression model

| **Patient** | **Episodes of peritonitis** | **Follow-up period** | | **Follow-up time (months)** | | | **Peritonitis status** | **Death** |
| --- | --- | --- | --- | --- | --- | --- | --- | --- |
|  |  | **Start date** | **End date** | **Start time** | **End time** | **Length of follow-up** |  |  |
| **Patients with peritonitis** | | |  |  |  |  |  |  |
| A | 0 | 13-May-2009 | 10-Jun-2010 | 0 | 13.0 | 13.0 | 0 | 0 |
| A | 1 | 11-Jun-2010 | 31-Dec-2013 | 13.01 | 45.6 | 42.6 | 1 | 0 |
| B | 0 | 21-Jul-2006 | 15-Oct-2006 | 0 | 2.8 | 2.8 | 0 | 0 |
| B | 1 | 16-Oct-2006 | 26-Dec-2006 | 2.81 | 5.1 | 2.3 | 1 | 0 |
| B | 2 | 27-Dec-2006 | 10-Oct-2007 | 5.11 | 14.5 | 9.4 | 1 | 0 |
| B | 3 | 11-Oct-2007 | 26-Aug-2008 | 14.51 | 25.0 | 10.5 | 1 | 1 |
| ... | ... | ... | ... | ... | **...** | ... | ... | ... |
| **Patients free of peritonitis** | | |  |  |  |  |  |  |
| C | 0 | 09-Dec-2009 | 31-Dec-2013 | 0 | 48.7 | 48.7 | 0 | 1 |

**Note:** For patient A, he experienced an episode of peritonitis on 11-Jun-2010, and he still survived at the end of the study (31-Dec-2013). Therefore, the immortal time for patient A is from 13-May-2009 to 10-Jun-2010. Patient B experienced 3 times of episode of peritonitis on 16-Oct-2006, 27-Dec-2006, and 11-Oct-2007, respectively, and experienced an death event on 26-Aug-2008. Similarly, the immortal time for patient B is from 21-Jul-2006 to 15-Oct-2006.

**Supplementary Table S4. The interactions between age and peritonitis for the assessment of the risk for** mortality

|  | HR (95% CI) | P value |
| --- | --- | --- |
| **All-cause mortality** |  |  |
| Peritonitis* | 2.70 (0.79-9.30) | 0.115 |
| Age (per year increase) | 1.06 (1.05-1.08) | <0.001 |
| Peritonitis*Age | 0.99 (0.98-1.01) | 0.578 |
| **Infection-related mortality** |  |  |
| Peritonitis* | 105.4 (3.81-2919.7) | 0.006 |
| Age ( per year increase) | 1.11 (1.06-1.15) | <0.001 |
| Peritonitis*Age | 0.95 (0.93-1.04) | 0.064 |
| **CV mortality** |  |  |
| Peritonitis* | 0.94 (0.15-5.76) | 0.944 |
| Age ( per year increase) | 1.06 (1.05-1.08) | <0.001 |
| Peritonitis*Age | 1.01 (0.98-1.04) | 0.473 |

*peritonitis was parameterized as a time-dependent covariate in the COX proportional hazards models.

**Supplementary Table S5. The HRs of peritonitis for all-cause and infection-related mortality progressively by year in the COX proportional hazards models**

|  | **Mean follow-up time (months )** | **No. of deaths** | **Crude HR (95% CI)** | **P value** | **Adjusted HR (95% CI)**^#^ | **P value** |
| --- | --- | --- | --- | --- | --- | --- |
| **All-cause mortality** | |  |  |  |  |  |
| ≤ 1 year | 11.3 ± 1.9 | 66 | 2.49 (1.40-4.44) | 0.054 | 1.12 (0.52-2.39) | 0.773 |
| ≤ 2 years | 20.7 ± 6.1 | 131 | 1.19 (0.75-1.88) | 0.465 | 0.80 (0.46-1.38) | 0.421 |
| ≤ 3 years | 27.7 ± 10.6 | 169 | 1.33 (0.92-1.93) | 0.130 | 1.00 (0.66-1.53) | 0.999 |
| ≤ 4 years | 32.0 ± 14.6 | 222 | 1.84 (1.37-2.47) | <0.001 | 1.51 (1.11-2.15) | 0.011 |
| ≤ 5 years | 34.3 ± 17.5 | 247 | 2.02 (1.53-2.66) | <0.001 | 1.74 (1.28-2.36) | <0.001 |
| ≤ 6 years | 35.5 ± 19.4 | 256 | 2.09 (1.60-2.74) | <0.001 | 1.84 (1.37-2.48) | <0.001 |
| ≤ 7 years | 35.9 ± 20.3 | 261 | 2.19 (1.68-2.85) | <0.001 | 1.95 (1.46-2.60) | <0.001 |
| ≤ 8 years | 36.0 ± 20.6 | 261 | 2.19 (1.68-2.85) | <0.001 | 1.95 (1.46-2.60) | <0.001 |
| **Infection-related mortality** | |  |  |  |  |  |
| ≤ 1 year | 11.3 ± 1.9 | 9 | 3.53 (0.91-13.70) | 0.068 | 0.57 (0.06-5.25) | 0.616 |
| ≤ 2 years | 20.7 ± 6.1 | 16 | 2.25 (0.79-6.43) | 0.129 | 1.06 (0.26-4.32) | 0.932 |
| ≤ 3 years | 27.7 ± 10.6 | 26 | 3.26 (1.48-7.16) | 0.003 | 1.79 (0.69-4.66) | 0.230 |
| ≤ 4 years | 32.0 ± 14.6 | 37 | 5.21 (2.69-10.09) | <0.001 | 4.08 (1.83-8.77) | 0.001 |
| ≤ 5 years | 34.3 ± 17.5 | 41 | 5.68 (3.01-10.71) | <0.001 | 4.44 (2.14-9.22) | <0.001 |
| ≤ 6 years | 35.5 ± 19.4 | 43 | 5.55 (2.98-10.33) | <0.001 | 4.46 (2.19-9.09) | <0.001 |
| ≤ 7 years | 35.9 ± 20.3 | 46 | 6.01 (3.27-11.04) | <0.001 | 4.94 (2.47-9.86) | <0.001 |
| ≤ 8 years | 36.0 ± 20.6 | 46 | 6.01 (3.27-11.04) | <0.001 | 4.94 (2.47-9.86) | <0.001 |

Note: HR, hazard risk; CI, confidence interval;

Peritonitis was parameterized as a time-dependent covariate.

^#^ Multivariable models were adjusted for age, sex, diabetes, history of CVD, 24h urine volume, hemoglobin, serum phosphorus, and serum albumin.
